# Highly efficient synthesis of the chiral ACE inhibitor intermediate (R)-2-hydroxy-4-phenylbutyrate ethyl ester via engineered bi-enzyme coupled systems

**DOI:** 10.1186/s40643-024-00814-z

**Published:** 2024-10-15

**Authors:** Yanmei Dai, Jinmei Wang, Zijuan Tao, Liangli Luo, Changshun Huang, Bo Liu, Hanbing Shi, Lan Tang, Zhimin Ou

**Affiliations:** 1 College of Pharmaceutical Science, Zhejiang University of Technology, Hangzhou, 310014 China; 2 College of Biological & Environmental Sciences, Zhejiang Wanli University, Ningbo, 315199 China; 3 Department of Respiratory Medicine, The Third Affiliated Hospital of Qiqihar Medical College, Qiqihar, China

**Keywords:** Carbonyl reductase, Bi-enzyme coupled, Fusion-expression, High-density fermentation, Substrate feeding strategy

## Abstract

(R)-2-Hydroxy-4-phenylbutyric acid ethyl ester ((R)-HPBE) is an essential chiral intermediate in the synthesis of angiotensin-converting enzyme (ACE) inhibitors. Its production involves the highly selective asymmetric reduction of ethyl 2-oxo-4-phenylbutyrate (OPBE), catalyzed by carbonyl reductase (CpCR), with efficient cofactor regeneration playing a crucial role. In this study, an in-situ coenzyme regeneration system was developed by coupling carbonyl reductase (CpCR) with glucose dehydrogenase (GDH), resulting in the construction of five recombinant strains capable of NADPH regeneration. Among these, the recombinant strain *E. coli* BL21-pETDuet-1-GDH-L-CpCR, where CpCR is fused to the C-terminus of GDH, demonstrated the highest catalytic activity. This strain exhibited an enzyme activity of 69.78 U/mg and achieved a conversion rate of 98.3%, with an enantiomeric excess (ee) of 99.9% during the conversion of 30 mM OPBE to (R)-HPBE. High-density fermentation further enhanced enzyme yield, achieving an enzyme activity of 1960 U/mL in the fermentation broth, which is 16.2 times higher than the volumetric activity obtained from shake flask fermentation. Additionally, the implementation of a substrate feeding strategy enabled continuous processing, allowing the strain to efficiently convert a final OPBE concentration of 920 mM, producing 912 mM of (R)-HPBE. These findings highlight the system’s improved catalytic efficiency, stability, and scalability, making it highly suitable for industrial-scale biocatalytic production.

## Graphical abstract

**Whole-cell biosynthesis of (R)-HPBE** Cost-effective and efficient synthesis of (R)-HPBE by constructing recombinant *E. coli* for in-situ coenzyme NADPH regeneration through fusion expression of carbonyl reductase CpCR and glucose dehydrogenase GDH in *E. coli*

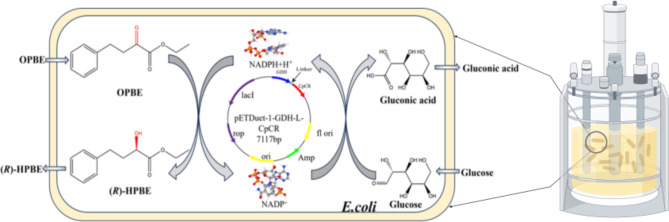

## Introduction

Chiral alcohols are of significant importance and utility in the synthesis of a diverse range of pharmaceuticals and specialty chemicals (Patel 2013). Of particular note is (R)-2-hydroxy-4-phenylbutyric acid ethyl ester, also known as (R)-HPBE, which serves as a crucial intermediate in the synthesis of angiotensin-converting enzyme (ACE) inhibitors (Oda et al. 1998; Xu and Ni 2015). Prily drugs include enalapril, benazepril, lisinopril, and other similar agents (Xu and Ni 2015), which are commonly used in the treatment of congestive heart failure and hypertension (Fröhlich et al. 2018). So far, a series of methods for the preparation of (R)-HPBE have been developed, including chemical multi-step synthesis (D’Arrigo et al. 2010; Lin et al. 2001), enzymatic asymmetric reduction of 2-oxo-4-phenyl-butyric acid ethyl ester (OPBE) (Su et al. 2020; Wang et al. 2015; Yuning et al. 2012) and kinetic resolution of racemate (Basetty et al. 2022; Liese et al. 2002). In recent years, the asymmetric reduction of OPBE to (R)-HPBE with recombinant carbonyl reductase as a catalyst has attracted considerable attention due to the advantages it offers in terms of high conversion rate, mild reaction conditions, green environmental protection and economic feasibility (Su et al. 2020; Wang et al. 2015; Yuning et al. 2012). Carbonyl reductase is available from a diverse range of microorganisms, including *Pseudomonas subtilis*, *Pseudomonas aeruginosa*, *Rhodococcus erythropolis* and *Aspergillus niger*, which can be employed as source donors for stereoselective carbonyl reductase (Kurbanoglu et al. 2007; Ni et al. 2013; Nie et al. 2007; Suwa et al. 2012; Wang et al. 2015; Ying et al. 2018). Carbonyl reductases with high stereoselectivity and enantioselectivity can catalyze highly selective asymmetric synthesis reactions of carbonyl compounds, thus providing an important method for the synthesis of chiral drugs and chiral drug intermediates with high optical purity (Chen et al. 2016).

As one of the oxidoreductases, carbonyl reductase requires the coenzyme NAD(H) or NADP(H) to transfer protons during its catalytic reaction (Fukuda et al. 2015, 2016; Li et al. 2017), Shen et al. used lyophilized *E. coli* cells carrying CgKR2 and lyophilized GDH crude enzyme powder for the preparation of (R)-HPBE, achieving an ideal enantiomeric excess (ee) value and conversion rate (99 and 100%, respectively) at 1 M OPBE (2012). In coenzyme-dependent enzyme reactions, the coenzyme plays a crucial role. However, the continuous consumption of coenzymes during the reaction can hinder the process, making coenzyme regeneration essential for maintaining the continuity of the catalytic reaction (Bachosz et al. 2023; Reetz 2012). Currently, the primary methods for coenzyme regeneration include enzymatic regeneration, such as glucose dehydrogenase (GDH) and formate dehydrogenase (FDH), electrochemical regeneration, and chemical regeneration using chemical reductants like hydrides (Mordhorst and Andexer 2020; Suryatin Alim et al. 2021). An in-vivo enzymatic coenzyme regeneration strategy, where coenzyme regeneration enzymes such as GDH, ADH, FDH, and LDH are integrated into engineered strains (Bachosz et al. 2023; Lin et al. 2022; Xu et al. 2007; Zhu et al. 2020), enables efficient and continuous regeneration of coenzymes. This significantly reduces the cost of coenzyme usage, simplifies processes, and minimizes by-product formation, making it more suitable for large-scale industrial applications and green production (Schrewe et al. 2013). There are two primary approaches to constructing in vivo coenzyme regeneration systems: co-expression and fusion expression. Co-expression involves the independent expression of both the target enzyme and the coenzyme regeneration enzyme within the same engineered strain (Kerrigan et al. 2011), these enzymes act independently within the same cell. For example, Yun et al. (2005) constructed a recombinant *E. coli* BL21 strain overexpressing YiaE from *E. coli* and GDH from *Bacillus subtilis*, achieving high product conversion rates and enantiomeric purity for (S)-HPBE (> 97% conversion rate, 98% e.e.). Fusion expression (Davis et al. 1999; Fang et al. 2015), on the other hand, involves the physical coupling of the target enzyme and the coenzyme regeneration enzyme into a multifunctional fusion enzyme through genetic engineering. This approach reduces the distance between the enzymes, minimizing transfer losses and significantly enhancing coenzyme regeneration efficiency. For instance, Torres Pazmiño et al. constructed a fusion protein linking Baeyer-Villiger monooxygenase (BVMO) with NADPH regeneration enzyme PTDH, which improved enantioselectivity and expanded the substrate range of the original BVMO (Pazmiño et al. 2008). Similarly, Kokorin et al. fused the gene for cytochrome P450 BM3 with FDH, resulting in up to a threefold increase in activity across multiple substrates (2021). These studies highlight the significant advantages of fusion enzymes in improving coenzyme regeneration efficiency and enhancing catalytic rates.

To maximize the potential of whole-cell catalysis, high-density fermentation (HDF) provides an ideal platform for achieving efficient biotransformation (Xiong et al. 2008a). Through precise environmental control, such as pH, oxygen concentration, and nutrient addition, cells can be maintained under optimal growth conditions (Kangwa et al. 2015). This level of control is unattainable in flask systems, as HDF allows for continuous monitoring and real-time adjustments, leading to higher cell densities (Xiong et al. 2008b). As a result, it has become the preferred method for producing biopharmaceuticals, enzymes, and biofuels (Shi et al. 2019). In continuous biocatalysis, substrate feeding strategies mitigate the inhibitory effects of high initial substrate concentrations on cell growth and enzyme activity (Hong 1986; Wang et al. 2021). They optimize substrate metabolism, balance metabolic loads, and prevent metabolic stress caused by excessive substrate uptake, ensuring that cells or enzymes do not accumulate undesirable byproducts (Zeng et al. 2018). Overall, the combination of high-density fermentation and optimized substrate feeding strategies provides ideal conditions for industrial-scale whole-cell catalysis, improving both product quality and yield while reducing production time.

This study established an in-vivo coenzyme regeneration system coupling CpCR and GDH, based on co-expression and fusion expression strategies. The kinetic constants of the CpCR-catalyzed reaction, conversion efficiency, enantiomeric selectivity, and stability of different systems were explored. After selecting the optimal recombinant strain, high-density fermentation was employed, followed by a substrate feeding strategy in continuous bioreactors to enhance substrate processing capacity. Ultimately, this approach enabled the efficient and stable synthesis of (R)-HPBE, demonstrating both economic viability and suitability for industrial-scale production.

## Materials and methods

### Bacterial strains

The bacterial strains and plasmids used in the study with their relevant characteristics are listed in Table 1. *E. coli* DH5α was used as the host for DNA manipulation and *E. coli* BL21 was used for recombinant protein expression and fermentation.

**Table 1:** Bacterial strains and plasmids used in this study

Recombinant strains	Description
*E. coli* BL21-pETDuet-1-CpCR	pETDuet-1, contain CpCR gene, Amp^R^
*E. coli* BL21-pET28a-CpCR	pET28a^+^, contain CpCR gene, Kan^R^
*E. coli* BL21-pACYCDuet-1-CpCR	pACYCDuet-1, contain CpCR gene, CmR^R^
*E. coli* BL21-pACYCDuet-1-GDH	pACYCDuet-1, contain GDH gene, CmR^R^
*E. coli* BL21-pETDuet-1-CpCR/pACYCDuet-1-GDH	pETDuet-1 contain CpCR gene, pACYCDuet-1 contain GDH gene, Kan^R^ and CmR^R^
*E. coli* BL21-pETDuet-1-CpCR-GDH	pETDuet-1, contain CpCR and GDH gene, Amp^R^
*E. coli* BL21-pETDuet-1-GDH-CpCR	pETDuet-1, contain CpCR and GDH gene, Amp^R^
*E. coli* BL21-pETDuet-1-CpCR-L-GDH	pETDuet-1, contain CpCR-linker-GDH gene, Amp^R^
*E. coli* BL21-pETDuet-1-GDH-L-CpCR	pETDuet-1, contain GDH-linker-CpCR gene, Amp^R^

### Construction of CpCR recombinant strains

**Fig. 1:**
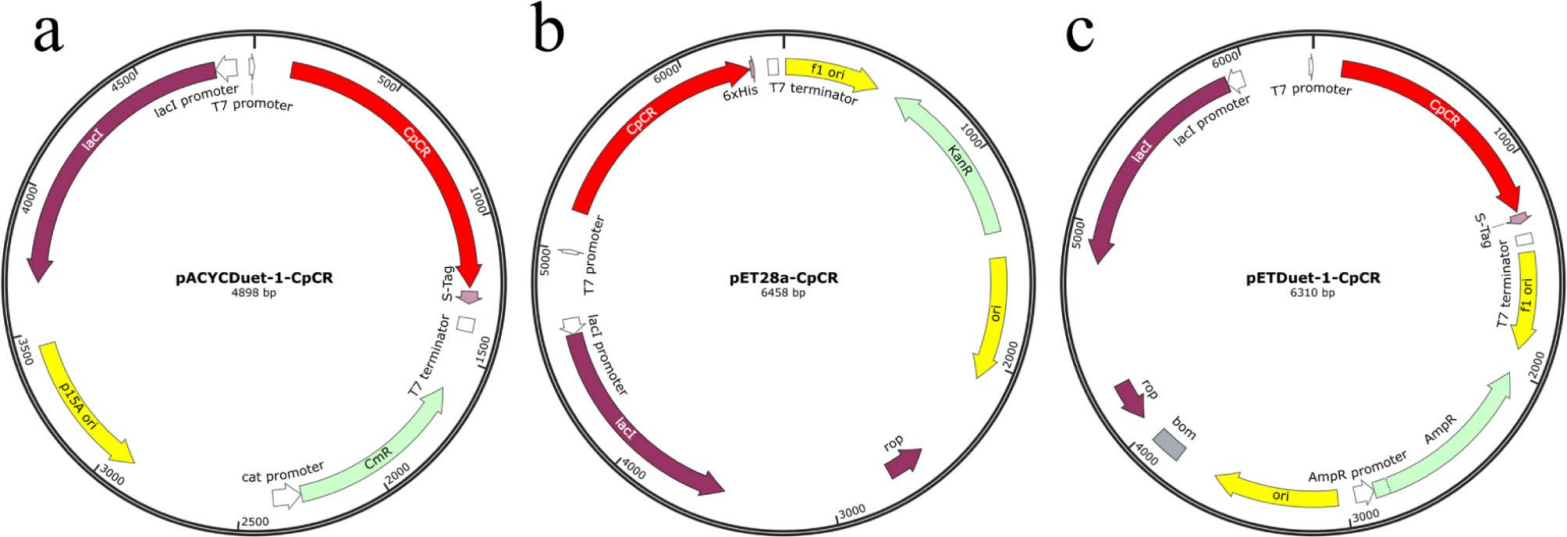
Structural diagram of the CpCR recombinant expression plasmids. (**a**), (**b**) and (**c**) stand for pACYCDuet-1-CpCR, pET28a-CpCR, pETDuet-1-CpCR

Using the whole genome of *Candida parapsilosis* ATCC 7330 as a template, PCR amplification was performed with primers CpCR1F (or CpCR2F) and CpCR1R (Table 2). The PCR products were verified by nucleic acid electrophoresis and successfully verified PCR products were then purified by gel extraction. The purified PCR products were ligated into plasmid vectors pre-digested with restriction enzymes Pst I and Xho I (or Sal I and Xho I using seamless cloning technology. The ligation products were transformed into *E. coli* DH5α, and recombinant plasmids were sequenced for verification. The correctly identified recombinant plasmids were designated as pETDuet-1-CpCR, pET28a-CpCR, and pACYCDuet-1-CpCR (Fig. 1). Finally, these plasmids were introduced into the *E. coli* BL21(DE3) host, resulting in the strains *E. coli* BL21-pETDuet-1-CpCR, *E. coli* BL21-pET28a-CpCR, and *E. coli* BL21-pACYCDuet-1-CpCR.

### Construction of a recombinant strains for the co-expression of CpCR and GDH recombinase proteins

**Fig. 2:**
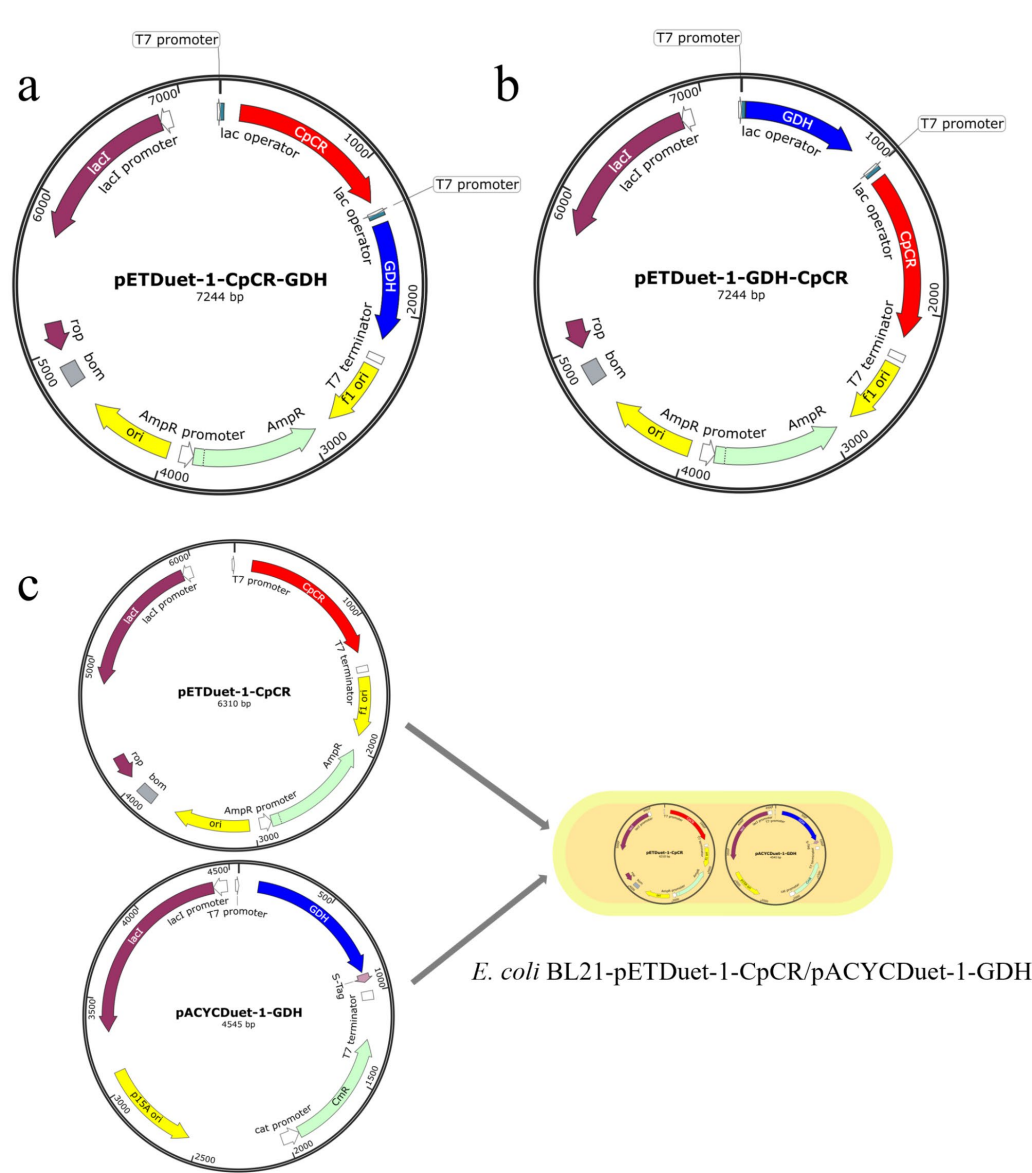
Plasmid map of the CpCR/GDH co-expressing recombinant strains. (**a**), (**b**) and (**c**) stand for pETDuet-1-CpCR-GDH, pETDuet-1-GDH-CpCR and pETDuet-1-CpCR/pACYCDuet-1-GDH

In order to express CpCR and GDH in *E. coli* BL21(DE3), the CpCR gene (GenBank: KC525950.1) was amplified from the whole genome of *Candida parapsilosis* ATCC 7330, while the GDH gene (GenBank: AAA22463.1) was amplified from the same source. The construction of recombinant strains and primer sequences are presented in Table 2. The CpCR and GDH fragments were purified, digested with PstI and XhoI, BamHI and XhoI respectively, and then cloned into pETDuet-1 and pACYCDuet-1 using the same restriction endonuclease to obtain pETDuet-1-CpCR and pACYCDuet-1-GDH. In order to co-express the CpCR and GDH genes in *E. coli* BL21, two expression vectors, pETDuet-1-CpCR and pACYCDuet-1-GDH, were transformed into *E. coli* BL21 (DE3) hosts. Recombinant strains exhibiting resistance to ampicillin and kanamycin were identified as those harboring plasmids expressing both CpCR and GDH. The successfully identified strain was designated as *E. coli* BL21-pETDuet-1-CpCR/pACYCDuet-1-GDH (Fig. 2c). CpCR was cloned behind the upstream T7 promoter of plasmid pETDuet-1, and GDH was cloned behind the downstream T7 promoter of pETDuet-1 (Fig. 2a), and the constructed recombinant plasmid was introduced into *E. coli* BL21(DE3) to obtain *E. coli* BL21-pETDuet-1-CpCR-GDH. GDH was cloned behind the upstream T7 promoter of plasmid pETDuet-1, and CpCR was cloned behind the downstream T7 promoter of pETDuet-1 (Fig. 2b) and transformed into *E. coli* BL21(DE3) in the same way to obtain *E. coli* BL21-pETDuet-1-GDH-CpCR.

**Table 2:** Primer sequences

Recombinant strains	Primers	Primers sequences	Restriction enzyme
*E.coli* BL21-pETDuet-1-CpCR	CpCR1F	AACTGCAGATGACTAAAGCAGTACCAGA	PstI
	CpCR1R	CCGCTCGAGAGCTTTGAATGCTTTGTCGA	XhoI
*E.coli* BL21-pET28a-CpCR	CpCR1F	AACTGCAGATGACTAAAGCAGTACCAGA	PstI
	CpCR1R	CCGCTCGAGAGCTTTGAATGCTTTGTCGA	XhoI
*E.coli* BL21-pACYCDuet-1-CpCR	CpCR2F	ACGGTCGACATGACTAAAGCAGTACCAGA	SalI
	CpCR1R	CCGCTCGAGAGCTTTGAATGCTTTGTCGA	XhoI
*E. coli* BL21-pETDuet-1-CpCR/pACYCDuet-1-GDH	CpCR1F	AACTGCAGATGACTAAAGCAGTACCAGA	PstI
	CpCR3R	ATAAGAATGCGGCCGCCTAAGCTTTGAAT	NotI
	GDH1F	GGAATTCCATATGATGTATCCGGATCTG	NdeI
	GDH1R	CCGCTCGAGACCACGACCCGCCTGAAA	XhoI
*E. coli* BL21-pETDuet-1-CpCR-GDH	CpCR1F	AACTGCAGATGACTAAAGCAGTACCAGA	PstI
	CpCR3R	ATAAGAATGCGGCCGCCTAAGCTTTGAAT	NotI
	GDH1F	GGAATTCCATATGATGTATCCGGATCTG	NdeI
	GDH1R	CCGCTCGAGACCACGACCCGCCTGAAA	XhoI
*E. coli* BL21-pETDuet-1-GDH-CpCR	GDH2F	AACTGCAG ATGTATCCGGATCTG	PstI
	GDH2R	ATAAGAATGCGGCCGCACCACGACCCGCCTGAAA	NotI
	CpCR4F	GGAATTCCATATGATGACTAAAGCAGTACCAGA	NdeI
	CpCR4R	CCGCTCGAGCTAAGCTTTGAAT	XhoI
*E. coli* BL21-pETDuet-1-CpCR-L-GDH	CpCR1F	AACTGCAGATGACTAAAGCAGTACCAGA	PstI
	CpCR-L-R	GGAACCTCCACCTCCGCTGCCTCCACCACCAGCTTTGAATGCTTTGTCG	None
	L-GDH-F	GGTGGTGGAGGCAGCGGAGGTGGAGGTTCCATGTATCCGGATCTG	None
	GDH1R	CCGCTCGAGACCACGACCCGCCTGAAA	XhoI
*E. coli* BL21-pETDuet-1-GDH-L-CpCR	GDH1F	GGAATTCCATATGATGTATCCGGATCTG	NdeI
	GDH-L-R	GGAACCTCCACCTCCGCTGCCTCCACCACCACCACGACCCGCCTGAAA	None
	L-CpCR-F	GGTGGTGGAGGCAGCGGAGGTGGAGGTTCCATGACTAAAGCAGTACCA	None
	CpCR1R	CCGCTCGAGAGCTTTGAATGCTTTGTCGA	XhoI

### Construction of recombinant strains for the fusion expression of CpCR and GDH recombinase proteins

**Fig. 3:**
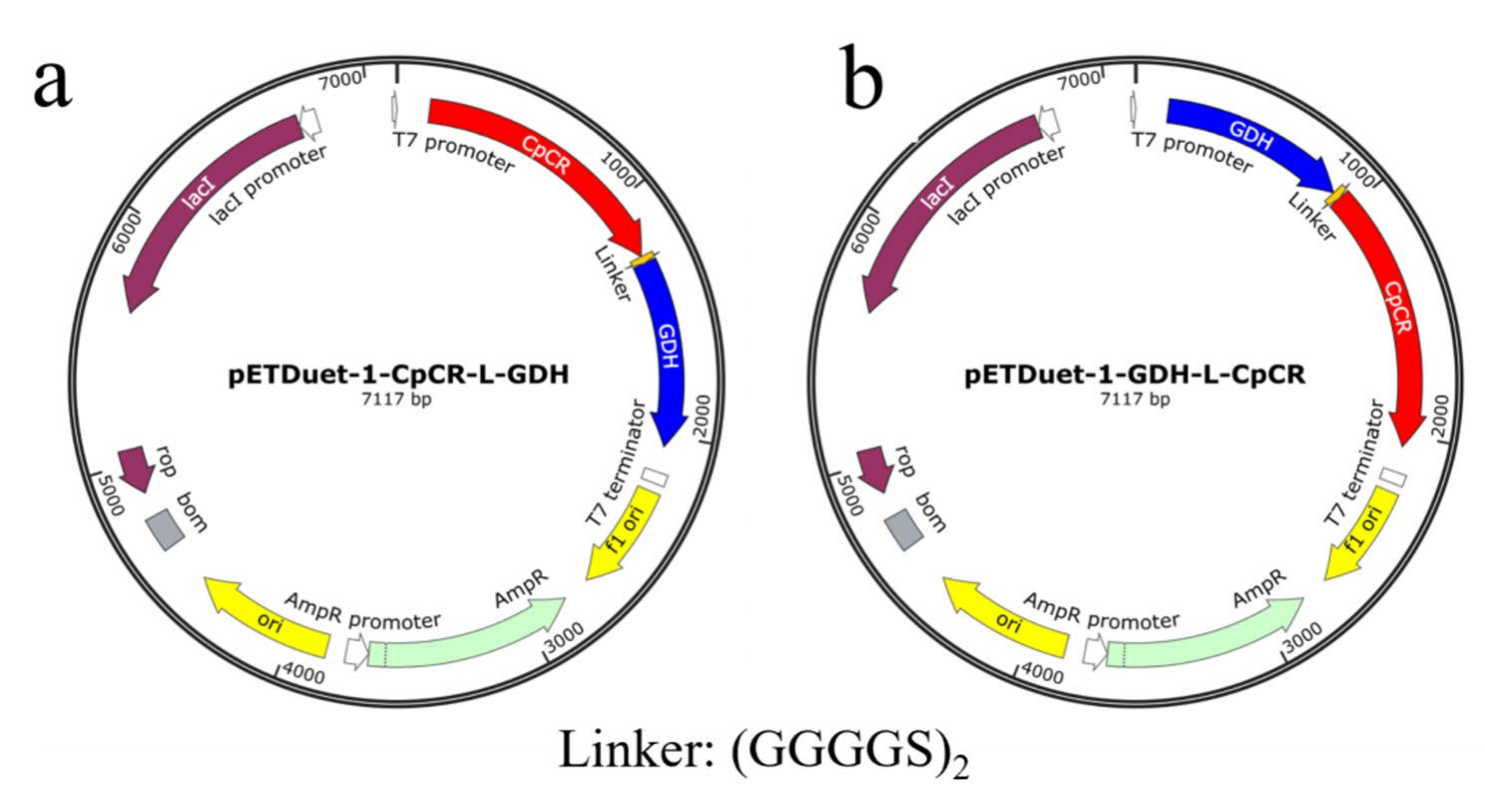
Plasmid map of the CpCR/GDH fusion expression recombinant strain. (**a**) and (**b**) stand for pETDuet-1-CpCR-L-GDH and pETDuet-1-GDH-L-CpCR

The primers were designed using Primer Premier 5 software, with the CpCR and GDH gene sequences serving as templates. GDH was fused to the C-terminus and N-terminus of the CpCR gene, and to maintain enzyme activity, a (GGGGS)₂ linker peptide was inserted between the two genes, resulting in the construction of the fusion genes CpCR-L-GDH and GDH-L-CpCR. The fusion expression genes were ligated to the expression vector pETDuet-1 using a seamless cloning kit, the ligation products were transformed into *E. coli* DH5α, and recombinant plasmids were sequenced for verification. The correctly identified recombinant plasmids were designated as pETDuet-1-CpCR-L-GDH, pETDuet-1-GDH-L-CpCR (Fig. 3). Finally, these plasmids were transformed into the *E. coli* BL21(DE3) host, resulting in the strains *E. coli* BL21- pETDuet-1-CpCR-L-GDH and *E. coli* BL21- pETDuet-1-GDH-L-CpCR.

### Biocatalyst preparation

#### Shake flask cultivation

*E. coli* was cultivated in Luria-Bertani (LB) medium at 37℃, while *Candida parapsilosis* ATCC 7330 was grown in Yeast Extract Peptone Dextrose (YPD) medium at 25℃. For recombinant selection, LB medium containing kanamycin (50 µg/mL) or ampicillin (50 µg/mL) or a combination thereof was employed.

For carbonyl reductase fermentation, a loopful of cells on a LB slant culture was inoculated into 50 mL of LB medium and incubated at 37℃ overnight. A 2% inoculum of cells was transferred to 150 mL of LB liquid medium and incubated until the optical density at 600 nm (OD600) reached 0.6–0.8. At this point, isopropyl β-D-thiogalactopyranoside (IPTG) was added at a final concentration of 1 mM, and the culture was further incubated for an additional 15 h.

#### High-density fermentation

Recombinant *E. coli* preserved in glycerol were inoculated into a conical flask containing 100 mL of LB medium and cultured at 37 °C with shaking at 180 rpm until the OD600 reached approximately 1.6, which was used as the seed culture. This seed culture was subsequently inoculated at 4% into a 5 L fermenter containing 2 L of fermentation medium (12.0 g/L yeast extract, 20.0 g/L peptone, 1.0 g/L NaCl, 6.0 g/L (NH₄)₂SO₄, 2.0 g/L MgSO₄·7 H₂O, 7.5 g/L glycerol, 1 mL/L trace elements, 3.0 g/L KH₂PO₄, 8.2 g/L Na₂HPO₄·12 H₂O, and 0.1% antifoam). The *E. coli* were cultured at 37 °C, 920 rpm, with aeration at 3 vvm, and the pH maintained at 7.0. A supplementation medium (60.0 g/L yeast extract, 100.0 g/L peptone, 10.0 g/L MgSO₄·7 H₂O, 500.0 g/L glucose) was added using a flow-through system, with 30% constant dissolved oxygen supplementation. When the OD600 reached 20, isopropyl-β-D-thiogalactopyranoside (IPTG) was added to a final concentration of 0.5 mM, and the culture was induced at 23 °C for 20 h.

### Enzyme activity and stability assays

The cultured cells were resuspended in 0.1 M phosphate buffer (pH 7.0), followed by cell disruption via ultrasonication. To obtain cell-free extracts, the resulting suspension was centrifuged to remove cell debris. The enzymatic activity of recombinant CpCR was determined by measuring the depletion of NADPH at 340 nm using spectrophotometry during the reduction of OPBE. The reaction mixture consisted of 100 mM phosphate buffer (pH 7.0), 1.0 mM OPBE, 1.0 mM NADPH, and an appropriate amount of enzyme or cell extract. One unit of enzyme activity was defined as the amount of enzyme required to oxidize 1 µmol of NADPH (for CpCR) or reduce 1 µmol of NADP⁺ (for GDH) per minute. Protein concentrations were measured using the Bradford method (Bradford 1976), with bovine serum albumin serving as the standard.

The purified fusion enzymes GDH-L-CpCR and CpCR-L-GDH, the co-expressed CpCR/GDH, and the single-enzyme CpCR were incubated at various temperatures (20, 30, 40, 50, 60, 70, 80, 90, and 100 °C) for 10 min, after which their residual enzymatic activity was measured to assess thermal stability. Additionally, the purified enzymes were incubated in buffer solutions at different pH levels (pH 4, 5, 6, 7, 8, and 9) at 25 °C for 1 h, and the residual activity was measured to evaluate pH stability. The purified enzyme was further incubated at 45 °C for 10, 20, 30, 40, 50, 60, 70, 80, 90, and 100 min, followed by measurement of residual activity, and a time stability curve was plotted based on the results.

### Bioreduction of OPBE to (R)-HPBE

#### Biocatalysis in shake flasks

The objective of this study was to investigate the effect of various parameters on the catalytic efficiency and productivity of isolated enzymes or whole microbial cells catalysing the asymmetric reduction of carbonyl compounds. The whole *E. coli* BL21-pETDuet-1-GDH-L-CpCR cells was employed as a catalyst, with the molar conversion rate of (R)-HPBE as an indicator. The parameters employed for the asymmetric reduction of OPBE to (R)-HPBE, including OPBE concentration, pH and glucose concentration (for NADPH regeneration), were individually optimised using a ‘one parameter at a time’ approach, whereby all parameters except the one under study were maintained at a constant level. The principal reaction conditions for the reduction of OPBE were established as follows (volume 10 ml): 10 mM OPBE, 0.1 g/ml wet cells, 50 g/L glucose and 0.1 mM NADP^+^ in 10 mM PB buffer (pH 7.0) at 30 °C for 24 h. Subsequently, the reaction mixture was subjected to centrifugation, after which the supernatant was extracted three times with ethyl acetate and subsequently dried with anhydrous MgSO_4_ for further GC analysis.

#### 5L fermenter continuous reaction

After the fermentation process was completed, the substrate OPBE and co-substrate glucose were added to the fermenter. The temperature was adjusted to 30 °C, and the pH was set to 7.5, with stirring maintained at 500 rpm for the subsequent biocatalysis. To overcome the inhibitory effects of high OPBE concentrations, a substrate feeding strategy was employed. Initially, 120 mM of OPBE was added at the start of the reaction. After two hours, continuous feeding of OPBE was implemented at a rate of 80 mM/h. After 10 h, the total substrate concentration reached 920 mM, at which point the substrate feed was stopped, allowing the reaction to continue until completion. This strategy effectively enhanced the production of (R)-HPBE.

### Analysis of OPBE and (R)-HPBE

The conversion rate and enantiomeric excess of (R)-HPBE were analyzed using a Shimadzu GC-2014 gas chromatograph. The conversion rate was defined as the ratio of the concentration of the converted substrate to the initial concentration of the substrate. The sample was analyzed using a gas chromatograph (GC) equipped with an Agilent J&W CP-Chirasil-Dex CB chiral column (Macherey-Nagel, 25 m × 0.25 mm, 0.25 μm). The temperatures of the injector, column, and FID were 250, 130, and 250 °C, respectively. The split ratio was 1:15. The flow rate was 2 mL/min. The retention times of OPBE, (R)-HPBE, and (S)-HPBE were 18.42, 25.48, and 26.49 min, respectively. The enantiomeric excess (e.e.%) value was calculated using the following equation: ${{\text{ee}}\% = \left[ {\left( {[{\text{R}}] - [{\text{S}}]} \right)/\left( {[{\text{R}}] + [{\text{S}}]} \right)} \right]}$. This value is expressed as a percentage (%).

## Results and discussion

### Screening of the optimal expression plasmid for CpCR

It is well-known that the pETDuet-1 plasmid features a unique dual promoter design, enabling the simultaneous expression of two target genes in *E. coli*. This characteristic is particularly advantageous for studying protein-protein interactions or the formation of multi-subunit complexes. The pET-28a plasmid, renowned for its strong T7 promoter and N-terminal His-tag, allows for efficient single-gene expression while simplifying subsequent protein purification processes. Additionally, the pACYCDuet-1 plasmid, with its low-copy p15A origin of replication and chloramphenicol resistance marker, is especially suited for co-expression studies when used alongside other high-copy plasmids, facilitating the expression of multiple genes without imposing an excessive burden on the host cell. Therefore, this study selected pETDuet-1, pET-28a, and pACYCDuet-1 as cloning hosts to investigate the optimal expression vector for CpCR in *E. coli*.

**Fig. 4:**
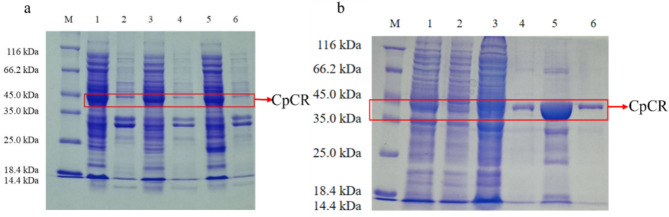
SDS-PAGE analysis of CpCR protein. (**a**) The SDS-PAGE analysis of CpCR expressed by *E. coli* BL21-pETDuet-1-CpCR, *E. coli* BL21-pET28a-CpCR and *E. coli* BL21-pACYCDuet-1-CpCR. (**b**) The purification of CpCR expressed by *E. coli* BL21-pETDuet-1-CpCR. ((**a**): Lane 1: Supernatant of *E. coli* BL21-pETDuet-1-CpCR; Lane 2: Precipitation of *E. coli* BL21-pETDuet-1-CpCR; Lane 3: Supernatant of *E. coli* BL21-pET28a-CpCR; Lane 4: Precipitation of *E. coli* BL21-pET28a-CpCR; Lane 5: Supernatant of *E. coli* BL21-pACYCDuet-1-CpCR; Lane 6: Precipitation of *E. coli* BL21-pACYCDuet-1-CpCR. (**b**): Lane 1: supernatant of *E. coli* BL21-pETDuet-1-CpCR breakage solution; Lane 2: precipitate of *E. coli* BL21-pETDuet-1-CpCR breakage solution; Lane 3: supernatant of concentrated breakage solution; Lane 4-Lane 6: eluent of protein CpCR purification)

The 1107 bp polynucleotide sequence, amplified from the genomic DNA of *Candida parapsilosis* ATCC 7330, represents a complete open reading frame that encodes a protein of 368 amino acid residues with a molecular weight of approximately 41 kDa. The nucleotide sequence of the *cpcr* gene has been deposited in GenBank under the accession number KC525950.1. Then *cpcr* was ligated into the MCS region of the expression vectors pETDuet-1, pET-28a, and pACYCDuet-1, successfully generating the recombinant strains *E. coli* BL21-pETDuet-1-CpCR, *E. coli* BL21-pET28a-CpCR, and *E. coli* BL21-pACYCDuet-CpCR. Following induction under optimal expression conditions, the wet cells were collected by centrifugation and lysed by an ultrasonic crusher, and subsequently analyzed for the target protein using SDS-PAGE. The SDS-PAGE results (Fig. 4a) revealed that the molecular weight of the expressed protein was approximately 41 kDa, closely aligning with the predicted molecular weight of CpCR based on the NCBI database. The cell-free extract from *E. coli* BL21-pETDuet-1-CpCR was concentrated and purified using a Ni-NTA column. The expression and purification were confirmed by SDS-PAGE. As shown in Fig. 4b, the His-tagged protein was successfully expressed and purified, producing a single band corresponding to the theoretical size of CpCR.

**Fig. 5:**
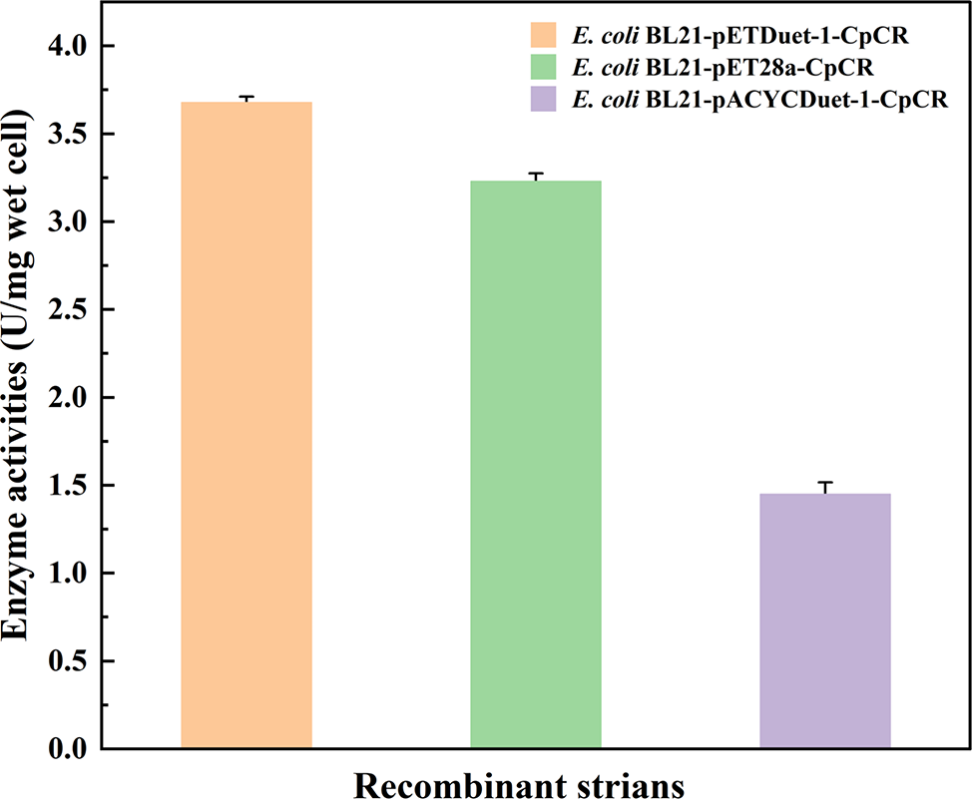
Enzyme activity of CpCR in different expression plasmids

Additionally, the enzyme activity of CpCR in each recombinant strain was measured in Fig. 5. The results demonstrated that the enzyme activities in *E. coli* BL21-pETDuet-1-CpCR, *E. coli* BL21-pET28a-CpCR, and *E. coli* BL21-pACYCDuet-1-CpCR were 3.68 U/mg wet cell, 3.23 U/mg wet cell, and 1.45 U/mg wet cell, respectively. These findings suggest that the pETDuet-1 plasmid is the most effective for CpCR expression, likely due to its high-copy nature compared to the low-copy pACYCDuet-1 plasmid.

### Protein expression in NADPH regeneration recombinant strains

Three recombinant *E. coli* strains co-expressing CpCR and GDH were constructed using two distinct strategies. One strategy involved a two-plasmid co-expression system, in which two genes were constructed in two plasmids. The other strategy employed a single-plasmid tandem co-expression system, in which two polyclonal sites were cloned in one plasmid. The three engineering strains were designated *E. coli* BL21-pETDuet-1-CpCR/pACYCDuet-1-GDH, *E. coli* BL21-pETDuet-1-CpCR-GDH, and *E. coli* BL21-pETDuet-1-GDH-CpCR, respectively. The N-terminus of CpCR was fused to the C-terminus of GDH, and the C-terminus of CpCR was fused to the N-terminus of GDH, altering the order of CpCR within the fusion proteins, and to ensure proper interaction between the two proteins without disrupting adjacent domains, a flexible linker peptide consisting of 10 amino acid residues, (GGGGS)₂, was inserted between them, resulting in the successful construction of two recombinant *E. coli* BL21 strains expressing fusion proteins: *E. coli* BL21-pETDuet-1-CpCR-L-GDH and *E. coli* BL21-pETDuet-1-GDH-L-CpCR.

**Fig. 6:**
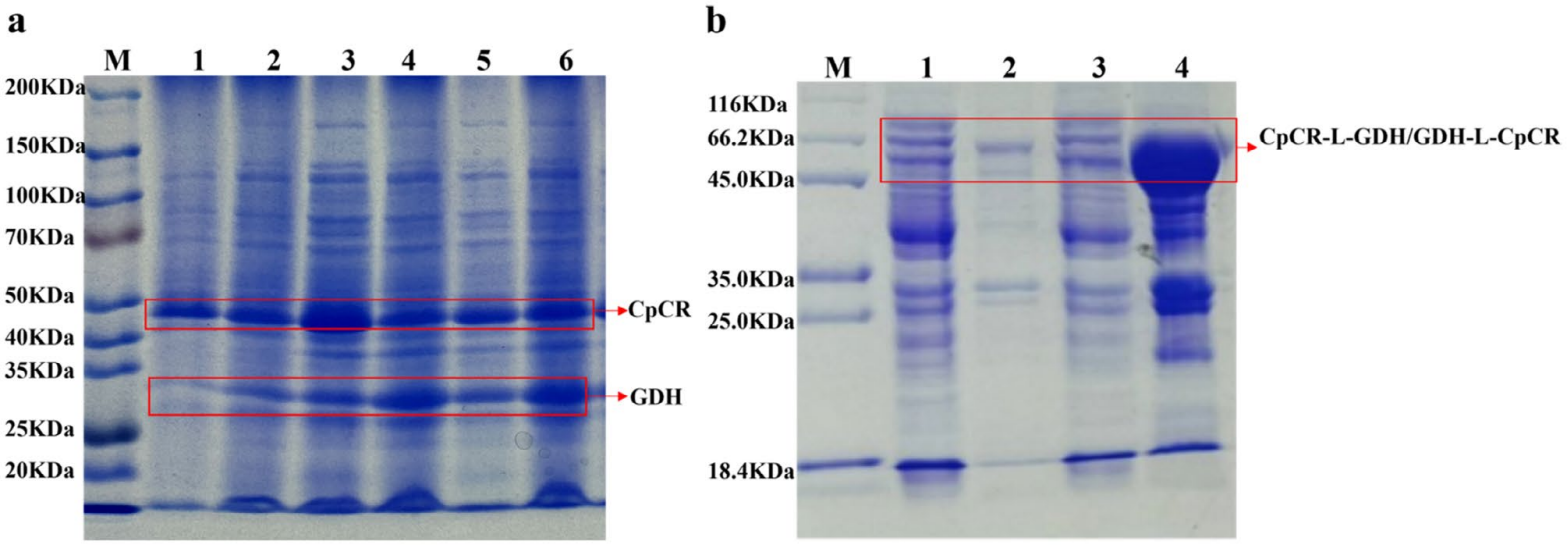
SDS-PAGE analysis of CpCR/GDH co-expression protein and fusion proteins CpCR-L-GDH and GDH-L-CpCR. (**a**) SDS-PAGE of CpCR/GDH co-expression (Lane 1: Supernatant of *E. coli* BL21-pETDuet-1-CpCR/pACYCDuet-1-GDH; Lane 2: Precipitation of *E. coli* BL21-pETDuet-1-CpCR/pACYCDuet-1-GDH; Lane 3: Supernatant of *E. coli* BL21-pETDuet-1-CpCR-GDH; Lane 4: Precipitation of *E. coli* BL21-pETDuet-1-CpCR-GDH; Lane 5: Supernatant of *E. coli* BL21-pETDuet-1-GDH-CpCR ; Lane 6: Precipitation of *E. coli* BL21-pETDuet-1-GDH-CpCR.); (**b**) DS-PAGE analysis of the fusion proteins CpCR-L-GDH and GDH-L-CpCR (Lane M: Protein Marker; lane 1: supernatant of pETDuet-1-CpCR-L-GDH; lane 2: precipitation of pETDuet-1-CpCR-L-GDH; lane 3: supernatant of pETDuet-1-GDH-L-CpCR; lane 4: precipitation of pETDuet-1-GDH-L-CpCR)

After inducing the expression of the recombinant strains, followed by ultrasonic disruption and centrifugation, both the precipitation and supernatant were collected separately for 12% SDS-PAGE analysis. Sequence analysis revealed that CpCR (1104 bp) encodes 368 amino acids with a predicted molecular weight of 41 kDa, while GDH (979 bp) encodes 260 amino acids with a predicted molecular weight of 28 kDa. Preliminary analysis of the cell-free extracts (Fig. 6a) indicates that the three co-expressed recombinant strains produced high levels of the expected proteins.

Figure 6b shows that the two fusion-expressing recombinant strains of CpCR and GDH produced a protein with the predicted size of 69 kDa, but its expression level was significantly lower. Most of the GDH-L-CpCR fusion enzyme (68.8 kDa) in *E. coli* BL21-pETDuet-1-GDH-L-CpCR was expressed in the form of inclusion bodies, with only minimal expression observed in the soluble form. This may be attributed to the rapid expression rate of the protein, which exceeds the cell’s folding capacity. The fusion protein, containing multiple domains, increases the complexity of the molecular structure, potentially making it more prone to misfolding.

### Study on the Kinetic Parameters of CpCR

**Table 3:** Kinetic parameters of CpCR in recombinant strains

Recombinant enzymes	Recombinant strians	Specific enzyme activity (U/mg)	Km (mM)	Kcat (s^− 1^)	Kcat/Km (mM/s)
CpCR	*E. coli* BL21-pETDuet-1-CpCR	16.7	19.32	1.77	0.091
	*E. coli* BL21-pET28a-CpCR	15.23	23.94	1.76	0.074
	*E. coli* BL21-pACYCDuet-1-CpCR	15.96	20.53	1.69	0.082
CpCR/GDH	*E. coli* BL21-pETDuet-1-CpCR/pACYCDuet-1-GDH	38.76	19.38	5.35	0.276
	*E. coli* BL21-pETDuet-1-CpCR-GDH	42.55	18.25	5.96	0.326
	*E. coli* BL21-pETDuet-1-GDH-CpCR	43.27	19.34	6.37	0.329
CpCR-L-GDH	*E. coli* BL21-pETDuet-1-CpCR-L-GDH	58.43	18.9	9.07	0.479
GDH-L-CpCR	*E. coli* BL21-pETDuet-1-GDH-L-CpCR	69.78	16.5	10.45	0.633

Table 3 summarizes the enzymatic kinetic parameters for all recombinant enzymes. Compared to the single-enzyme CpCR, the co-expression of CpCR and GDH resulted in significantly improved catalytic efficiency, as evidenced by higher specific enzyme activity, increased Kcat values, and overall enhanced catalytic performance. This enhancement in enzymatic activity is primarily due to the role of GDH in regenerating NADPH. The fusion expression enzymes exhibit significant advantages in enzymatic kinetics compared to both the co-expression and single-expression enzymes. Specifically, compared to the single enzyme CpCR expressed by *E. coli* BL21-pET28a-CpCR (15.23 U/mg) and the co-expressed dual enzymes CpCR/GDH from the strain *E. coli* BL21-pETDuet-1-CpCR/pACYCDuet-1-GDH (38.76 U/mg), the fusion enzyme GDH-L-CpCR, with CpCR located at the C-terminus, exhibited significantly higher specific enzyme activity (69.78 U/mg). Fusion enzymes also display superior catalytic efficiency, with a Kcat of 10.45 s⁻¹ in GDH-L-CpCR, significantly outperforming both the single enzyme CpCR expressed by *E. coli* BL21-pET28a-CpCR (1.76 s⁻¹) and the co-expressed dual enzymes CpCR/GDH from the strain *E. coli* BL21-pETDuet-1-CpCR/pACYCDuet-1-GDH (5.35 s⁻¹). Furthermore, the fusion expression strains maintain a lower Km, such as 16.5 mM in *E. coli* BL21-pETDuet-1-GDH-L-CpCR, indicating better substrate affinity compared to both single- and co-expression strains. Finally, the Kcat/Km values of fusion enzymes, such as 0.633 mM/s in GDH-L-CpCR, demonstrate superior catalytic efficiency, significantly exceeding that of the single-expression strains and co-expression enzymes. These results highlight that fusion expression system not only outperform single- and co-expression systems but also offer enhanced synergistic interactions between enzymes, making them ideal for industrial applications requiring high catalytic efficiency. Compared to CgKR (Km: 0.1 mM) (Shen et al. 2012), all recombinant CpCR enzymes exhibit higher Km values, indicating lower substrate affinity. Although CpCR demonstrates lower affinity than CgKR, under specific conditions, the co-expression of both enzymes in close spatial proximity reduces the need to add separate enzymes to the reaction system, these results suggest that a well-designed fusion expression strategy can significantly enhance the catalytic performance of enzymes, providing potential options for further industrial applications.

### Whole-cell synthesis of (R)-HPBE by recombinant strains expressing the CpCR enzyme

**Fig. 7:**
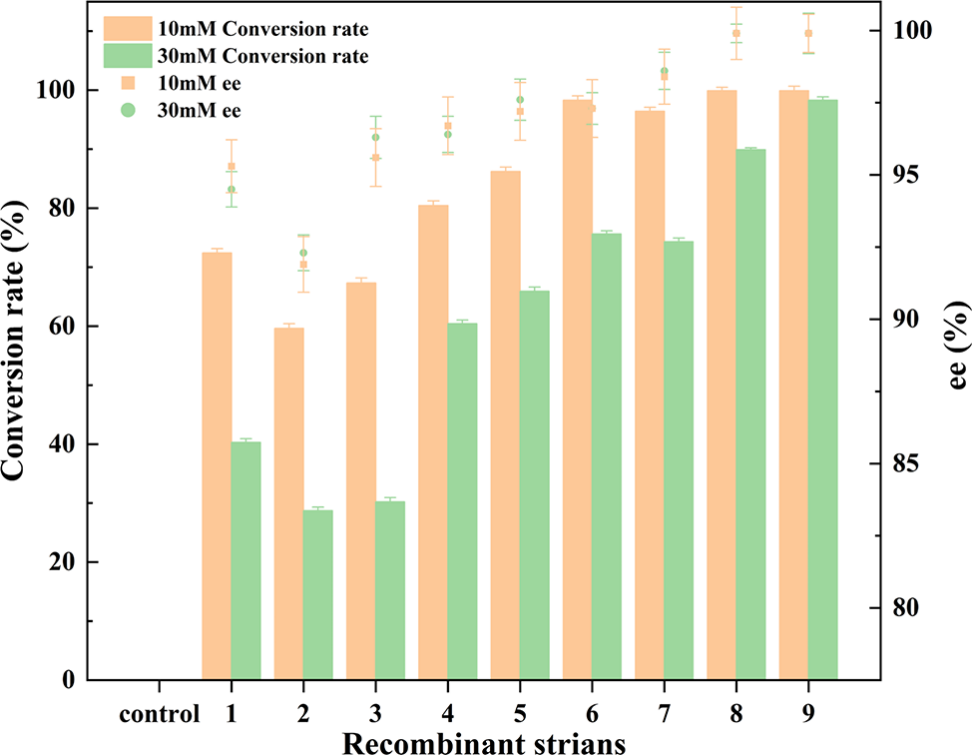
Conversion rates and enantiomeric excess (ee%) of (R)-HPBE by different recombinant strains. (Control: reaction without cells; 1: *E. coli* BL21-pETDuet-1-CpCR; 2: *E. coli* BL21-pACYCDuet-1-CpCR; 3: *E. coli* BL21-pET28a-CpCR; 4:combination of *E. coli* BL21-pETDuet-1-CpCR and *E. coli* pACYCDuet-1-GDH; 5: *E. coli* BL21-pETDuet-1-CpCR/pACYCDuet-1-GDH; 6: *E. coli* BL21-pETDuet-1-CpCR-GDH; 7: *E. coli* BL21-pETDuet-1-GDH-CpCR; 8: *E. coli* BL21-pETDuet-1-CpCR-L-GDH; 9: *E. coli* BL21-pETDuet-1-GDH-L-CpCR)

The eight CpCR recombinant strains constructed above were used for whole-cell biocatalysis to synthesize (R)-HPBE according to the described protocol, with a cell-free substrate reaction system serving as the control. Additionally, a reaction catalyzed by two recombinant strains, *E. coli* BL21-pETDuet-1-CpCR and *E. coli* pACYCDuet-1-GDH, was performed to evaluate the advantage of co-expressing CpCR and GDH in a single host. The reactions were carried out at substrate concentrations of 10 mM and 30 mM for 12 h. The biocatalytic results are shown in Fig. 7. Strain 9 (*E. coli* BL21-pETDuet-1-GDH-L-CpCR) exhibited the highest conversion rates at both 10 mM and 30 mM substrate concentrations, reaching 99.9% and 98.3%, respectively, demonstrating a strong synergistic effect between GDH and CpCR when linked by a flexible linker. Strain *E. coli* BL21-pETDuet-1-CpCR-L-GDH also achieved conversion rates of 99.9% at 10 mM and 89.9% at 30 mM. The difference between these two strains suggests that the order of GDH and CpCR, as well as the nature of the linker, affects the efficiency of enzyme cooperation.

In contrast, strains expressing only CpCR (1: *E. coli* BL21-pETDuet-1-CpCR, 2: *E. coli* BL21-pET28a-CpCR, and 3: *E. coli* BL21-pACYCDuet-1-CpCR) exhibited conversion rates below 75% at both 10 mM and 30 mM substrate concentrations, with a notable drop to below 65% at 30 mM. This may be attributed to insufficient NADPH regeneration at higher substrate concentrations, which limits the catalytic efficiency of CpCR. Reaction 4 involved a mixed reaction of separately expressed *E. coli* BL21-pETDuet-1-CpCR and *E. coli* BL21-pACYCDuet-1-GDH, achieving a conversion rate of about 80.43% at 10 mM, which dropped to approximately 60.4% at 30 mM. Although both enzymes functioned together, their synergistic effect was weaker than that observed in strains with co-expression or fusion expression in a single host, likely due to the absence of spatial or mechanistic interaction between the enzymes. Strains 5, 6, and 7 co-expressed CpCR and GDH, among these, strain 6 (*E. coli* BL21-pETDuet-1-CpCR-GDH) showed relatively high conversion rates of 98.3% at 10 mM and 75.6% at 30 mM. However, compared to the fusion enzymes, the co-expressed enzymes exhibited moderate conversion rates, possibly because GDH and CpCR coexist but do not interact as closely as in the fusion enzymes.

In terms of stereoselectivity (ee value), all strains maintained high ee values, indicating that the different strategies had little impact on the stereoselectivity of CpCR. Strain 9 (*E. coli* BL21-pETDuet-1-GDH-L-CpCR) achieved an ee value of 99% under both 10 mM and 30 mM substrate concentrations, demonstrating that the fusion of GDH and CpCR not only enhanced the conversion efficiency but also preserved the high enantioselectivity of CpCR.

The different expression strategies had a significant impact on the conversion rate and ee values of CpCR and GDH. The fusion expression of GDH and CpCR, particularly in the recombinant strain *E. coli* BL21-pETDuet-1-GDH-L-CpCR, where CpCR is located at the C-terminus, resulted in a marked improvement in both conversion rate and stereoselectivity. Notably, the fusion-expressing strains also exhibited the higher substrate tolerance. Regardless of whether the substrate concentration was 10 mM or 30 mM, the fusion-expressing strains maintained extremely high catalytic efficiency. This indicates that the fusion of GDH and CpCR greatly enhanced their synergistic interaction, enabling these strains to sustain efficient reactions even at high substrate concentrations.

### Stability of recombinant proteins

**Fig. 8:**
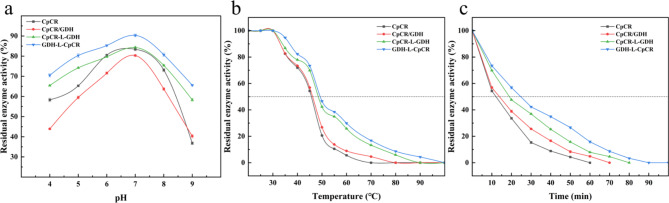
Residual enzyme activity of purified enzymes CpCR, CpCR/GDH, CpCR-L-GDH, and GDH-L-CpCR after incubation under different conditions. **a**, **b**, and **c** stand for the pH stability, thermal stability and time-dependent stability

The stability of three different enzyme expression systems—CpCR single enzyme, CpCR/GDH co-expression, and CpCR-GDH fusion enzymes (including two fusion orientations: CpCR-L-GDH and GDH-L-CpCR)—was evaluated under various incubation conditions, using residual enzyme activity as the primary measure (Fig. 8). The stability of these systems was assessed across a range of pH levels, temperatures, and incubation times.

In the pH stability tests (Fig. 8a), all enzyme expression systems showed maximum residual activity at pH 7. At this optimal pH, the GDH-L-CpCR fusion enzyme exhibited the highest residual activity at 90.3%, followed by CpCR-L-GDH with 84.3%. The CpCR single enzyme and CpCR/GDH co-expression enzyme displayed lower maximum activities, retaining 83.4% and 80.3%, respectively. Under more extreme pH conditions (pH 4 and pH 9), all systems experienced a reduction in residual activity, but the fusion enzymes demonstrated greater tolerance. Both fusion constructs retained more than 50% activity at pH 4 and pH 9, while the single enzyme and co-expression systems dropped to around 40%. This indicates that the fusion expression of CpCR with GDH significantly enhances pH stability, particularly for GDH-L-CpCR, which maintained the highest activity across a broader pH range.

Similar trends were observed in thermal stability tests (Fig. 8b). GDH-L-CpCR exhibited the best thermal stability, retaining 82.3% residual activity at 40 °C and 29.9% at 60 °C. CpCR-L-GDH showed slightly lower thermal stability, retaining 77.9% at 40 °C and 25.8% at 60 °C. The CpCR single enzyme lost significant activity at higher temperatures, with only 71.9% residual activity at 40 °C and almost complete inactivation at 60 °C. The CpCR/GDH co-expression enzyme displayed intermediate thermal stability, retaining 73.4% residual activity at 40 °C but declining sharply at higher temperatures. These results demonstrate that fusion expression significantly improves thermal stability, with GDH-L-CpCR being the most robust, especially at elevated temperatures.

In the time-dependent stability tests conducted at 45 °C (Fig. 8c), the fusion enzymes demonstrated superior long-term stability. GDH-L-CpCR retained 42.3% residual activity after 30 min of incubation, indicating enhanced stability under thermal stress. Similarly, CpCR-L-GDH maintained 36.9% residual activity after the same incubation period. In contrast, the CpCR single enzyme and co-expression systems exhibited significantly faster declines, with residual activities of only 15.3% and 25.6%, respectively, after 30 min. These results suggest that fusion expression not only improves short-term thermal stability but also extends enzyme longevity during prolonged high-temperature incubation.

In summary, under all tested conditions, the fusion enzyme systems, particularly GDH-L-CpCR, consistently exhibited superior stability. These systems demonstrated the highest residual activity in terms of pH stability, thermal resistance, and long-term activity retention at elevated temperatures. These findings highlight the potential of fusion enzyme strategies to significantly improve enzyme performance and durability, making them well-suited for industrial applications that demand robust and stable enzymes across a broad range of operational conditions.

### Optimization of Fusion enzyme GDH-L-CpCR induction expression conditions

To further increase the soluble protein expression and activity of the recombinant strain *E. coli* BL21-pETDuet-1-GDH-L-CpCR, the protein expression under different induction conditions was studied. Enzyme activity in the cells was measured to determine the optimal expression conditions for maintaining protein functionality.

**Fig. 9:**
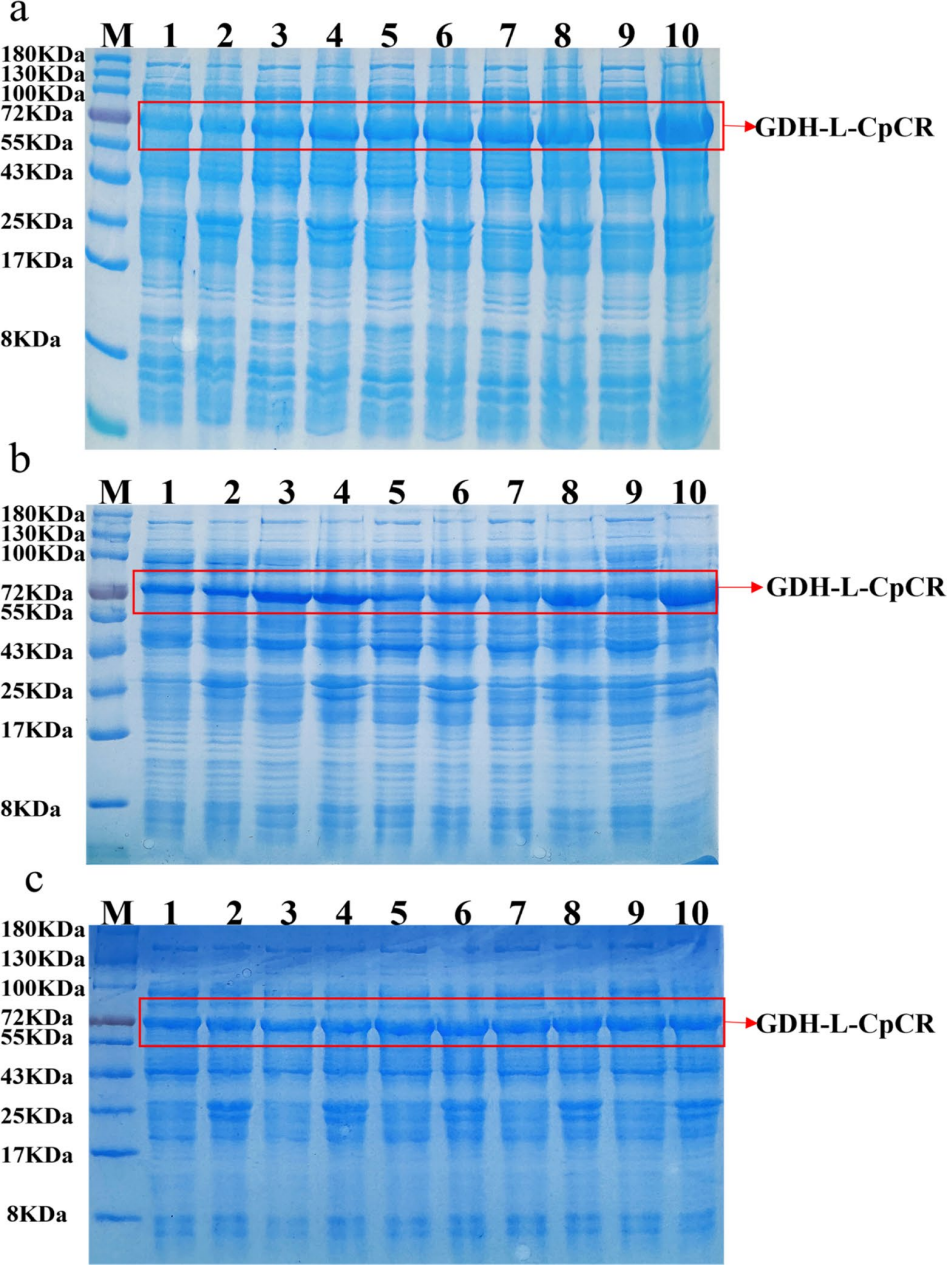
SDS-PAGE of GDH-L-CpCR in *E. coli* BL21-pETDuet-1-GDH-L-CpCR at different induction conditions. (**a**) IPTG concentrations. Lane 1, 3, 5, 7, and 9 show supernatant from cell extracts with 0.1 mM, 0.3 mM, 0.5 mM, 0.7 mM, and 0.9 mM IPTG. Lanes 2, 4, 6, 8, and 10 show the corresponding precipitation; (**b**) Temperatures. Lane 1, 3, 5, 7, and 9 represent the supernatant of cell-free extracts induced at 18, 23, 28, 30, and 37 °C, respectively. Lane 2, 4, 6, 8, and 10 show the corresponding precipitation at the same induction temperatures; (**c**) Times. Lane 1, 3, 5, 7, and 9 show the supernatant of cell-free extracts after induction times of 8, 12, 16, 20, and 24 h, respectively. Lane 2, 4, 6, 8, and 10 depict the corresponding pellets for the same induction times

Figure 9 illustrates the expression of the fusion protein GDH-L-CpCR under different IPTG concentrations, induction temperatures, and induction times. In Fig. 9a, the band intensity of the target protein increases with higher IPTG concentrations, reaching its peak at 0.5 mM IPTG (lane 5) in the supernatant. Higher IPTG concentrations lead to a gradual increase in inclusion body formation, indicating that 0.5 mM IPTG is the most effective concentration for balancing protein yield and solubility. Figure 9b evaluates the effect of induction temperature on protein expression. At lower temperatures (lanes 1, 3, 5, 7, and 9 in the supernatant), protein expression is relatively low. As the temperature increases, both soluble and insoluble protein expression are enhanced. There is little difference in protein expression levels between 23, 28, and 30 °C, but at 37 °C (lanes 9 and 10), the intensity of the target protein band in the supernatant decreases, and inclusion body formation becomes more prominent. This suggests that higher temperatures result in misfolding and aggregation of the GDH-L-CpCR fusion protein. Thus, 23–28 °C is considered the optimal temperature range for producing soluble GDH-L-CpCR. Figure 9c shows the protein expression levels at various induction times. SDS-PAGE analysis reveals that the strongest band for soluble GDH-L-CpCR is observed after 16 h of induction (lane 5). Beyond 16 h (lanes 7 and 9), the protein begins to accumulate in the precipitation. Therefore, 16 h of induction is optimal for maximizing soluble protein yield while minimizing the formation of inclusion bodies.

**Fig. 10:**
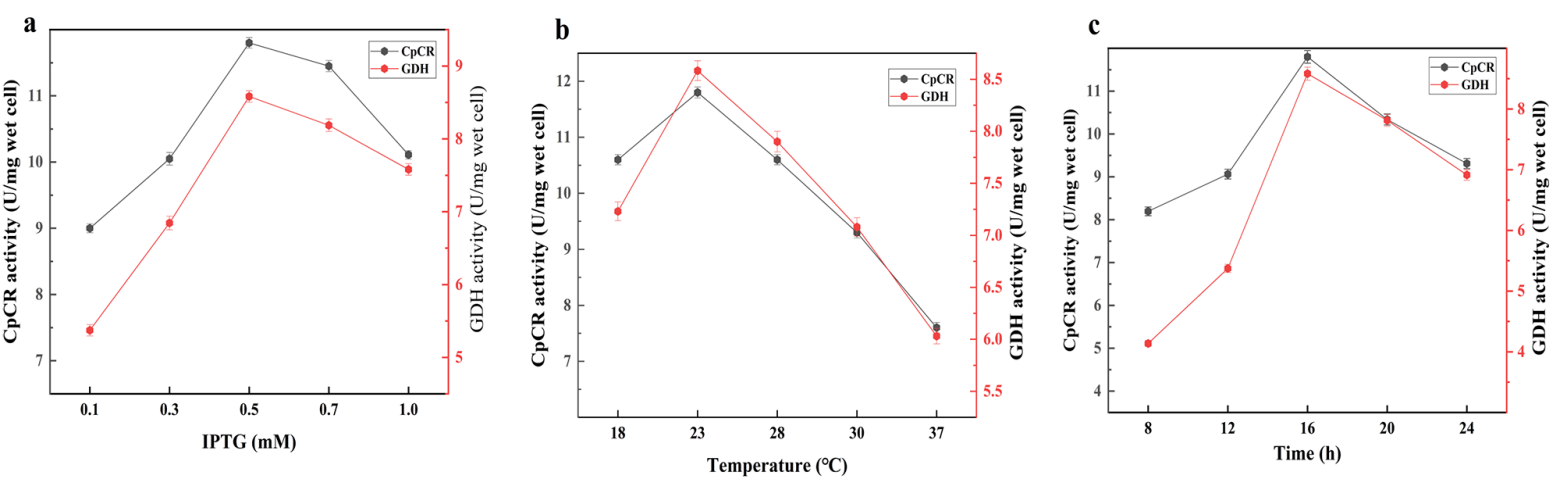
Optimization of enzyme induction conditions. (**a**), (**b**), and (**c**) represent the effects of IPTG concentration, induction temperature, and induction time, respectively, on the enzyme activity of the fusion protein GDH-L-CpCR

Figure 10 shows the enzyme activities of GDH and CpCR for the fusion protein GDH-L-CpCR under various induction conditions. The optimal enzyme activity was observed at an IPTG concentration of 0.5 mmol/L, an induction time of 16 h, and an induction temperature of 23 °C. Under these conditions, the enzyme activity of *E. coli* BL21-pETDuet-1-GDH-L-CpCR reached 11.8 U/mg wet cell for CpCR and 8.582 U/mg wet cell for GDH.

Based on the SDS-PAGE analysis and enzyme activity measurements of the fusion protein GDH-L-CpCR under various induction conditions, the optimal parameters for the expression of soluble and active GDH-L-CpCR were determined to be 0.5 mM IPTG, 23 °C, and 16 h of induction.

### Optimization of reaction conditions for Fusion expression strain *E. coli* BL21-pETDuet-1-GDH-L-CpCR

**Fig. 11:**
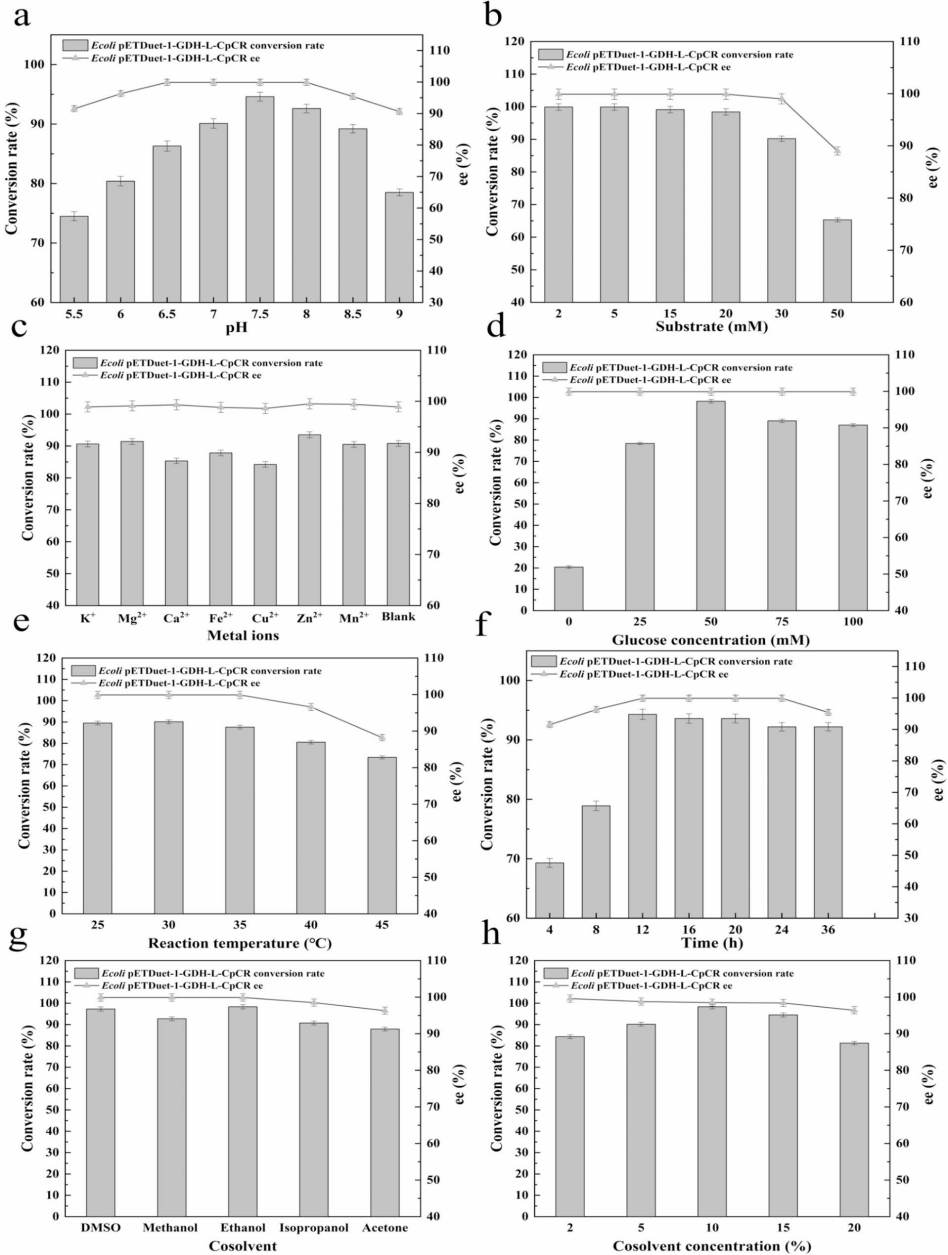
Optimization of Whole-Cell Catalytic Conditions for Strain *E. coli* BL21-pETDuet-1-GDH-L-CpCR. (**a**) pH; (**b**) Substrate concentration; (**c**) Metal ions; (**d**) Glucose concentration; (**e**) Temperature; (**f**) Time; (**g**) Cosolvent; (**h**) Cosolvent concentration

The impact of pH on the catalytic efficiency of fusion-expressing strain was examined in the range of 5.5 to 9.0. As illustrated in Fig. 11a, the conversion rate exhibited a gradual increase with the elevation of pH between pH 5.5 and 7.5. The recombinant strain *E. coli* BL21-pETDuet-1-GDH-L-CpCR, which exhibited the highest fusion expression, catalyzed the conversion rate of OPBE into (R)-HPBE at pH 7.5, with a conversion rate of (R)-HPBE reaching 94.6% and an ee% value reaching 99.9%. The conversion rate of OPBE exhibited a decline when the pH exceeded 8.0, accompanied by a discernible impact on the ee% of (R)-HPBE. This phenomenon may be attributed to the alteration in pH levels, which has the potential to influence the stereo conformation of the enzyme active site. The conversion rate trend indicates that the enzyme is most active in a weak base environment and exhibits the highest ee% of (R)-HPBE. Consequently, 100 mM pH 7.5 PB was identified as the optimal buffer for reduction reaction.

The effect of substrate concentration (2 mM, 5 mM, 10 mM, 15 mM, 20 mM, 30 mM and 50 mM) on the asymmetric catalytic reduction reaction using the fusion-expressing recombinant engineered bacterium was then examined. As illustrated in Fig. 11b, the conversion rate with *E coli.* BL21-pETDuet-1-GDH-L-CpCR reached 99.9% at the substrate concentration of 15 mM. When the substrate concentration exceeded 30 mM, a certain degree of decrease in the conversion rate was observed, with a conversion rate of 90.2%. At a substrate concentration of 50 mM, the conversion rate was 65.2%, although the ee value appeared to be significantly altered. It can be observed that the inhibition of the reaction is particularly evident when the substrate concentration exceeds 30 mM. Furthermore, the ee value appears to be influenced by the high concentration of OPBE.

Metal ions typically participate in the stabilization of active sites, substrate binding, and the formation of reaction intermediates. In oxidoreductases, metal ions often serve as electron transfer centers, influencing cellular and enzymatic catalytic reactions through mechanisms such as structural stability, substrate binding, charge neutralization, and electron transfer functions. For example, K⁺ plays a role in maintaining cellular osmotic balance; Mg²⁺ acts as a cofactor for many enzymes, particularly in ATP-dependent reactions, where it is crucial for energy transfer; Ca²⁺ is involved in cell signaling and membrane stability, regulating cell growth and division; additionally, Fe²⁺ and Cu²⁺ are essential elements in redox reactions, participating in the respiratory chain and regulating enzymatic activity; Zn²⁺ and Mn²⁺ function as metal cofactors, ensuring proper protein folding and activity, with Zn²⁺ also being a key component of zinc finger proteins. These ions play a central role in biocatalytic reactions, and both their deficiency and excess can significantly affect enzymatic performance and normal cellular metabolism (Dudev and Lim 2014). Therefore, this study investigates the effects of different metal ions on the biocatalytic activity of the fusion expression strain *E. coli* BL21-pETDuet-1-GDH-L-CpCR. The effects of seven metal cations such as K^+^, Mg^2+^, Ca^2+^, Fe^2+^, Cu^2+^, Zn^2+^, and Mn^2+^ on the fusion-expressing strain *E. coli* BL21-pETDuet-1-GDH-L-CpCR were investigated. As illustrated in Fig. 11c, the presence of various metal ions influenced the whole-cell catalytic performance of the fusion enzyme. The conversion rates were consistently above 80% for most metal ions tested, except for Fe²⁺ and Cu²⁺, which exhibited a clear inhibitory effect. Among the tested ions, Zn²⁺ displayed a higher conversion rate compared to the control, maintaining conversion rates of 95%, which could be attributed to its role as a cofactor, stabilizing the enzyme structure without interfering with the catalytic site. Conversely, Cu²⁺ had the most pronounced inhibitory effect, reducing the conversion rate to approximately 85%. The inhibitory effect of Cu²⁺ might be explained by its interaction with cysteine residues in the active site of CpCR, leading to conformational changes that reduce the enzyme’s catalytic efficiency. Despite the inhibitory effects of certain metal ions on the enzyme’s conversion rate, the high enantiomeric excess (ee) maintained across all conditions suggests that the presence of metal ions does not affect the enzyme’s stereoselectivity. This indicates that while metal ions may interfere with the enzyme’s overall catalytic efficiency, they do not alter the enzyme’s ability to produce the desired chiral product.

Glucose plays a pivotal role in the cascade enzyme-catalysed reaction system, serving as an auxiliary substrate that facilitates coenzyme regeneration. As illustrated in Fig. 11d, the concentration of the auxiliary substrate exerts a significant influence on the biocatalytic efficiency of the fusion enzyme. As the concentration of glucose increased, the conversion rate also increased gradually. Upon reaching a glucose concentration of 50 g/L, the conversion rate of *E. coli* BL21-pETDuet-1-GDH-L-CpCR reached 92.2%, accompanied by an ee value of 99.9%. As the glucose concentration continued to increase, the conversion rate decreased, yet the ee value remained largely unaltered. Consequently, the optimal auxiliary substrate concentration was determined to be 50 g/L.

The effect of temperature on the fusion enzyme activity is clearly evident in Fig. 11e, with enzyme activity exhibiting a relatively significant response to temperature fluctuations. Within a certain temperature range of 25–35 °C, the conversion rates achieved by fusion-expressed recombinant strain were found to be largely consistent. The highest conversion rate of 90.2% was observed by *E. coli* BL21-pETDuet-1-GDH-L-CpCR at 30℃. When the temperature exceeded 35 ℃, the fusion enzyme activity decreased and the ee value was also affected. The high temperature would lead to partial inactivation of the enzyme, so the optimum temperature for the reaction of the fusion enzyme-catalysed reaction system was 30 ℃.

The conversion rate and enantiomeric excess (ee%) of (R)-HPBE catalyzed by the recombinant strain *E. coli* BL21-pETDuet-1-GDH-L-CpCR were analyzed over a range of reaction times (4 h, 8 h, 12 h, 16 h, 20 h, 24 h, 36 h) (Fig. 11f). Initially, both the conversion rate and ee% increased rapidly within the first 4 h, indicating high catalytic activity and stereoselectivity. From 4 to 8 h, the conversion rate continued to rise, albeit at a slower pace, while the ee% remained consistently above 99.5%. The optimal reaction time was determined to be approximately 12 h, at which point the conversion rate reached a maximum of 94.3%, and the ee% was 99.9%. Beyond 12 h, no significant changes in either conversion rate or ee% were observed. These results suggest that 12 h is the optimal time to achieve maximum catalytic efficiency and enantioselectivity, as extending the reaction time beyond this point neither improves the conversion rate nor enhances enantioselectivity, but instead unnecessarily prolongs the process.

Cosolvents play a dual role in both cellular and enzymatic catalytic reactions. On the one hand, the appropriate type and concentration of cosolvent can enhance substrate solubility, improve enzyme stability, and increase reaction selectivity, thereby enhancing overall reaction efficiency. On the other hand, excessive concentrations of cosolvent may lead to enzyme inactivation or cellular damage (Yang et al. 2014). To identify the most suitable cosolvent for the CpCR-catalyzed synthesis of (R)-HPBE, the effects of dimethyl sulfoxide (DMSO), methanol, ethanol, isopropanol, and acetone were analyzed. The results, shown in Fig. 11g, indicate that among the five hydrophilic organic solvents tested, DMSO and ethanol exhibited the highest conversion rates with minimal negative impact on the enantiomeric excess of (R)-HPBE. In particular, ethanol was identified as the optimal cosolvent for *E. coli* BL21-pETDuet-1-GDH-L-CpCR, achieving a conversion rate of 97.4%. Further investigation into the effect of ethanol concentration on the asymmetric synthesis of (R)-HPBE by the recombinant strain revealed that at an ethanol concentration of 10%, the conversion rate of *E. coli* BL21-pETDuet-1-GDH-L-CpCR reached a maximum of 98.3% (Fig. 11h). However, when the ethanol concentration exceeded 10%, the conversion rate began to decline, likely due to ethanol’s inhibitory effect on intracellular enzyme activity or normal cellular metabolism. In contrast, variations in ethanol concentration had minimal impact on the ee% of (R)-HPBE. Therefore, 10% ethanol was determined to be the optimal cosolvent for this reaction system.

In summary, we optimized the reaction conditions for the catalytic bioconversion of OPBE to (R)-HPBE using fusion-expressing recombinant strain *E. coli* BL21-pETDuet-1-GDH-L-CpCR. Under the optimal conditions, reaction temperature of 30 °C, reaction system pH of 7.5, auxiliary substrate glucose addition at 50 g/L, reaction time of 12 h, and the addition of 10% ethanol, the fusion-expressing recombinant strain *E. coli* BL21-pETDuet-1-GDH-L-CpCR achieved a conversion rate of 98.3% for 30 mM OPBE, with an enantiomeric excess (ee) value of 99.9%.

### Biocatalysis under substrate feeding strategy in a 5 L fermentor

**Fig. 12:**
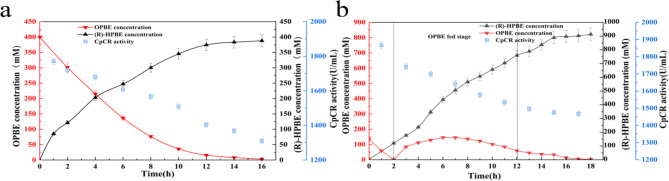
Time course of batch and fed-batch bioconversion of OPBE to (R)-HPBE by the strain *E. coli* BL21-pETDuet-1-GDH-L-CpCR. (**a**) Batch bioconversion; (**b**) Fed-batch bioconversion

To further increase the substrate processing capacity of OPBE and enhance the yield of (R)-HPBE, a 5 L fermenter was used to culture the recombinant strain *E. coli* BL21-pETDuet-1-GDH-L-CpCR. The fermenter employed a high-density fermentation strategy, using a fed-batch system with continuous nutrient feeding under constant dissolved oxygen conditions. This approach achieved higher cell density and increased enzymatic activity. Results showed that the enzyme activity reached 1960 U/mL after high-density fermentation, significantly higher than the 121 U/mL achieved in shake-flask fermentation, representing a 16.2-fold improvement in volumetric enzyme activity.

A comparison was made between two strategies: one-time substrate addition and substrate feeding. In the batch reaction (Fig. 12a), OPBE was added to the reactor at an initial concentration of 400 mM. After 16 h of catalytic reaction, the (R)-HPBE yield reached 387 mM, and the enzyme activity in the fermentation broth was 1309 U/mL. In contrast, the substrate feeding strategy involved the gradual addition of OPBE, maintaining a lower substrate concentration and preventing enzyme inhibition caused by high substrate levels. After 17 h of catalytic reaction, the enzyme activity remained 1467 U/mL. Throughout the process, the OPBE concentration was kept below 150 mM, and the total substrate processed reached 920 mM—a 1.3-fold increase compared to the batch reaction. The (R)-HPBE yield reached 912 mM and the space-time yield reached 644mM /(L·day), which is 2.2 times higher than that achieved in the batch process (Fig. 12b). These results demonstrate that the substrate feeding strategy can be effectively applied in high-concentration carbonyl reduction reactions catalyzed by the recombinant strain *E. coli* BL21-pETDuet-1-GDH-L-CpCR. By employing this strategy, not only was the substrate processing capacity significantly increased, but product yield was also substantially improved. Moreover, the strategy optimizes enzymatic catalytic efficiency within the fermenter, providing an efficient and convenient in-situ catalytic method for industrial-scale production.

## Conclusion

This study based on previous work in which the carbonyl reductase gene *cpcr* from *C. parapsilosis* ATCC 7330 was cloned. The gene was individually cloned into the expression vectors pETDuet-1, pET28a, and pACYCDuet-1, and the effects of different vectors on the enzymatic activity of carbonyl reductase CpCR were evaluated. The recombinant strain *E. coli* BL21-pETDuet-1-CpCR exhibited significantly higher enzyme activity (3.68 U/mg wet cell) compared to the strains *E. coli* BL21-pET28a-CpCR (3.23 U/mg wet cell) and *E. coli* BL21-pACYCDuet-1-CpCR (1.45 U/mg wet cell). Therefore, the pETDuet-1 vector was identified as the optimal vector for cpcr gene expression.

In the process of catalytic reduction of carbonyl compounds, carbonyl reductase equires a coenzyme to function as a hydrogen or electron donor. Due to the high cost of coenzymes, there is a strong need to reduce expenses while improving the conversion efficiency of CpCR-catalyzed reactions. This study presents a highly efficient coenzyme regeneration system, developed through both co-expression and fusion expression, which effectively addresses the challenge of coenzyme regeneration. In this study, five recombinant *E. coli* strains expressing different forms of CpCR and GDH were successfully constructed using two distinct strategies: fusion expression and co-expression. The fusion expression strains included *E. coli* BL21-pETDuet-1-CpCR-L-GDH (with CpCR at the N-terminus) and *E. coli* BL21-pETDuet-1-GDH-L-CpCR (with CpCR at the C-terminus). Additionally, three co-expression strains were developed: the dual-plasmid strain *E. coli* BL21-pETDuet-1-CpCR/pACYCDuet-1-GDH and two single-plasmid strains, *E. coli* BL21-pETDuet-1-CpCR-GDH and *E. coli* BL21-pETDuet-1-GDH-CpCR. The primary difference between the single-plasmid co-expression strains lies in the position of the *cpcr* gene, with the former positioned upstream of the T7 promoter and the latter downstream. The enzymatic kinetics of the five recombinant enzymes with coupled cofactor regeneration by GDH, as well as three single-enzymes CpCR, were evaluated. Compared to the single-enzymes CpCR, the recombinant proteins co-expressed and fused with GDH exhibited significantly higher specific enzyme activity and Kcat values due to the efficient regeneration of the cofactor NADPH. The fusion enzymes, in particular, demonstrated enhanced activity due to the spatial proximity of the active sites, which facilitated direct substrate channeling. The recombinant enzyme GDH-L-CpCR, with CpCR at the C-terminus, showed the highest catalytic efficiency, with a specific enzyme activity of 69.78 U/mg and a Kcat/Km ratio of 0.633 mM/s.

Next, we tested the whole-cell catalytic efficiency of the recombinant strains in converting OPBE to (R)-HPBE. At a substrate concentration of 10 mM, all strains with cofactor regeneration by GDH achieved conversion rates above 80% and ee values exceeding 95%. In contrast, the single-enzymes CpCR achieved only 60–70% conversion rate, although the ee values were similar. When the substrate concentration was increased to 30 mM, the fusion strain *E. coli* BL21-pETDuet-1-GDH-L-CpCR demonstrated the highest catalytic efficiency (98.3%) and ee value (99.9%). This indicates that the recombinant strains with NADPH regeneration greatly improved catalytic efficiency by overcoming cofactor limitations. Additionally, the *E. coli* BL21-pETDuet-1-GDH-L-CpCR fusion strain, with its spatially connected active sites, promoted substrate channeling and simplified enzyme regulation, resulting in a significant increase in conversion rates.

Furthermore, the stability of the single-enzymes CpCR, co-expressed CpCR/GDH, and the fusion enzymes CpCR-L-GDH and GDH-L-CpCR was evaluated. The results indicated that the fusion enzymes exhibited superior stability in terms of pH tolerance, thermal stability, and long-term stability compared to both the single CpCR and co-expressed CpCR/GDH enzymes. The GDH-L-CpCR fusion enzyme showed the highest resistance to acidic and basic conditions, as well as improved thermal and temporal stability, likely due to the spatial proximity of its active sites, which formed a more compact structure. This tight fusion reduced the surface exposure of sensitive regions, particularly under extreme conditions.

We next optimized the protein expression and catalytic conditions of the fusion-expressing recombinant strain *E. coli* BL21-pETDuet-1-GDH-L-CpCR, which showed the highest conversion efficiency. Based on cell enzyme activity assays and SDS-PAGE analysis, the optimal expression conditions were determined to be 0.5 mM IPTG, an induction time of 16 h, and a temperature of 23 °C. For the catalytic reaction conditions, the optimal parameters for whole-cell catalysis of OPBE to (R)-HPBE by *E. coli* BL21-pETDuet-1-GDH-L-CpCR were found to be a reaction temperature of 30 °C, pH 7.5, with the addition of 50 g/L glucose as the auxiliary substrate, 3 mM Zn²⁺, 10% ethanol, and a reaction time of 12 h. Under these conditions, the strain achieved a conversion rate of 98.3% for 30 mM OPBE, with an enantiomeric excess (ee) value of 99.9%.

To further increase the substrate processing capacity of *E. coli* BL21-pETDuet-1-GDH-L-CpCR, high-density fermentation was performed using a 5 L fermenter. Compared to shake-flask cultures (121 U/mL), high-density fermentation yielded an enzyme activity of 1960 U/mL, representing a 16.2-fold increase in volumetric enzyme activity. Based on these results, a substrate feeding strategy was implemented in the 5 L bioreactor for whole-cell catalysis, where OPBE was continuously fed into the system. This approach resulted in a substrate processing capacity of 920 mM OPBE, yielding 912 mM of (R)-HPBE. The combination of high-density fermentation and substrate feeding strategy significantly enhanced (R)-HPBE production, offering a viable process for large-scale biocatalytic production.

This study demonstrates the unique catalytic efficiency and stability advantages of the dual-enzyme coupling system. The fusion enzyme GDH-L-CpCR exhibits remarkable industrial potential due to its efficient NADPH regeneration mechanism and substrate channeling effect, which significantly improve catalytic efficiency while minimizing substrate diffusion losses. Furthermore, the GDH-L-CpCR fusion enzyme possesses excellent thermal stability, pH tolerance, and long-term operational stability, making it suitable for complex industrial conditions. The combination of high-density fermentation and substrate feeding strategy significantly increased the enzyme activity of GDH-L-CpCR, ensuring adequate substrate supply and avoiding substrate inhibition. This approach not only maximized catalytic efficiency but also optimized the space-time productivity. Overall, this study provides a simplified process with improved production efficiency, offering broad industrial application prospects.

## Data Availability

All data that support the findings of this study are included within this paper.

## Abbreviations

(R)-HPBE: (R)-2-Hydroxy-4-phenylbutyric acid ethyl ester
ACE: Angiotensin-converting enzyme
OPBE: 2-oxo-4-phenyl-butyric acid ethyl ester
CpCR: Carbonyl reductase
NADPH: Nicotinamide adenine nucleotide phosphate
GDH: Glucose dehydrogenase
E. coli: Escherichia coli
ee%: Enantiomeric excess%
NADP^+^: Nicotinamide Adenine Dinucleotide Phosphate
FDH: Formate dehydrogenase
ADH: Alcohol dehydrogenase
LDH: Lactate dehydrogenase
HDF: High-density fermentation
BVMO: Baeyer-Villiger monooxygenase
YPD: Yeast extract peptone dextrose
LB medium: Luria–Bertani medium
